# (±)-4,12,15,18,26-Penta­hydroxy-13,17-dioxahepta­cyclo­[14.10.0.0^3,14^.0^4,12^.0^6,11^.0^18,26^.0^19,24^]hexa­cosa-1,3(14),6(11),7,9,15,19,21,23-nona­ene-5,25-dione methanol disolvate

**DOI:** 10.1107/S1600536814006643

**Published:** 2014-04-02

**Authors:** Maayan Gil, Joseph Almog, Faina Dubnikova, Benny Bogoslavski, Shmuel Cohen

**Affiliations:** a The Institute of Chemistry, The Hebrew University of Jerusalem, Jerusalem 91904, Israel

## Abstract

The title compound, C_24_H_14_O_9_·2CH_3_OH, displays a chair-shaped form. The two di­hydro­indenone ring systems are located above and below the central fused-ring system, the dihedral angles between the mean planes of di­hydro­indenone ring systems and the mean plane of central fused-ring system are 67.91 (5) and 73.52 (4)°, respectively. In the crystal, extensive O—H⋯O hydrogen bonds, weak C—H⋯O hydrogen bonds and C—H⋯π inter­actions link the mol­ecules into a three-dimensional supra­molecular architecture.

## Related literature   

For an isomer of the title compound possessing a cup-shaped form, see: Mahmood *et al.* (2011 ▶). For a related structure, see: Almog *et al.* (2009 ▶).
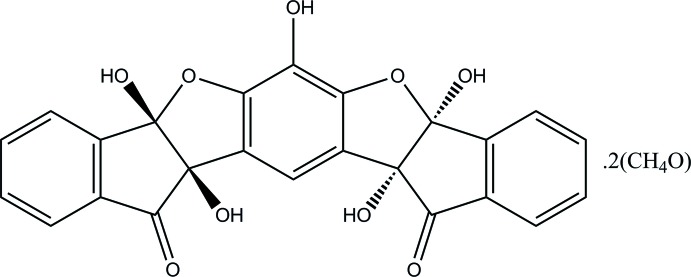


## Experimental   

### 

#### Crystal data  

C_24_H_14_O_9_·2CH_4_O
*M* *_r_* = 510.44
Triclinic, 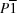

*a* = 8.8243 (13) Å
*b* = 10.3974 (16) Å
*c* = 14.348 (2) Å
α = 72.936 (3)°
β = 74.639 (2)°
γ = 75.792 (3)°
*V* = 1193.3 (3) Å^3^
*Z* = 2
Mo *K*α radiation
μ = 0.11 mm^−1^
*T* = 295 K
0.20 × 0.13 × 0.08 mm

#### Data collection  

Bruker SMART CCD area-detector diffractometer
13982 measured reflections
5549 independent reflections
3395 reflections with *I* > 2σ(*I*)
*R*_int_ = 0.050

#### Refinement  

*R*[*F* ^2^ > 2σ(*F* ^2^)] = 0.067
*wR*(*F* ^2^) = 0.153
*S* = 1.01
5549 reflections
345 parameters
2 restraints
H atoms treated by a mixture of independent and constrained refinement
Δρ_max_ = 0.30 e Å^−3^
Δρ_min_ = −0.33 e Å^−3^

### 

Data collection: *SMART* (Bruker, 2007 ▶); cell refinement: *SAINT* (Bruker, 2007 ▶); data reduction: *SAINT*; program(s) used to solve structure: *SHELXS97* (Sheldrick, 2008 ▶); program(s) used to refine structure: *SHELXL97* (Sheldrick, 2008 ▶); molecular graphics: *ORTEP-3 for Windows* (Farrugia, 2012 ▶); software used to prepare material for publication: *SHELXL97*.

## Supplementary Material

Crystal structure: contains datablock(s) I, global. DOI: 10.1107/S1600536814006643/xu5780sup1.cif

Structure factors: contains datablock(s) I. DOI: 10.1107/S1600536814006643/xu5780Isup2.hkl

Supporting information file. DOI: 10.1107/S1600536814006643/xu5780Isup3.cml

CCDC reference: 993652

Additional supporting information:  crystallographic information; 3D view; checkCIF report

**Fig. 3.**
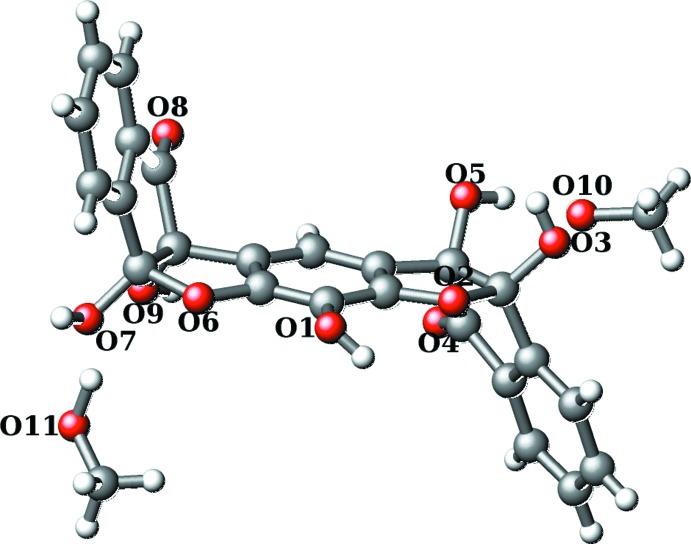
Enhanced figure.

Enhanced figure: interactive version of Fig. 3

## Figures and Tables

**Table 1:** Hydrogen-bond geometry (Å, °) *Cg* is the centroid of the C1–C6 benzene ring.

*D*—H⋯*A*	*D*—H	H⋯*A*	*D*⋯*A*	*D*—H⋯*A*
O1—H1*O*⋯O8^i^	0.82	1.92	2.702 (2)	160
O3—H3*O*⋯O5^ii^	0.82	1.91	2.725 (2)	171
O5—H5*O*⋯O10	0.82	1.80	2.610 (3)	171
O7—H7*O*⋯O1^iii^	0.82	2.14	2.916 (2)	158
O9—H9*O*⋯O4^iv^	0.82	1.95	2.751 (2)	166
O10—H10*O*⋯O11^iv^	0.82 (1)	1.85 (2)	2.650 (4)	163 (5)
O11—H11*O*⋯O7	0.83 (1)	2.31 (3)	3.010 (3)	142 (4)
O11—H11*O*⋯O9	0.83 (1)	2.15 (3)	2.873 (3)	145 (4)
C18—H18⋯O6^iii^	0.93	2.56	3.370 (3)	146
C21—H21⋯O1^v^	0.93	2.57	3.231 (3)	129
C11—H11⋯*Cg* ^vi^	0.93	2.63	3.501 (3)	156

Symmetry codes: (i) 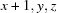; (ii) 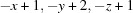; (iii) 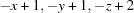; (iv) 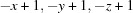; (v) 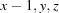; (vi) 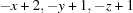.

**Fig. 1.**
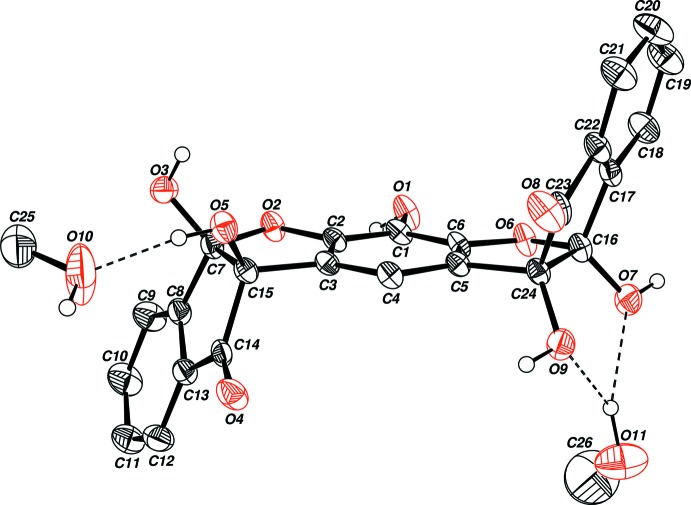
ORTEP drawing of (I) dimethanolate with the atom-numbering scheme. Displacement ellipsoids are drawn at the 50% probability level. For the sake of clarity only hydroxyl H atoms are shown.

**Fig. 2.**
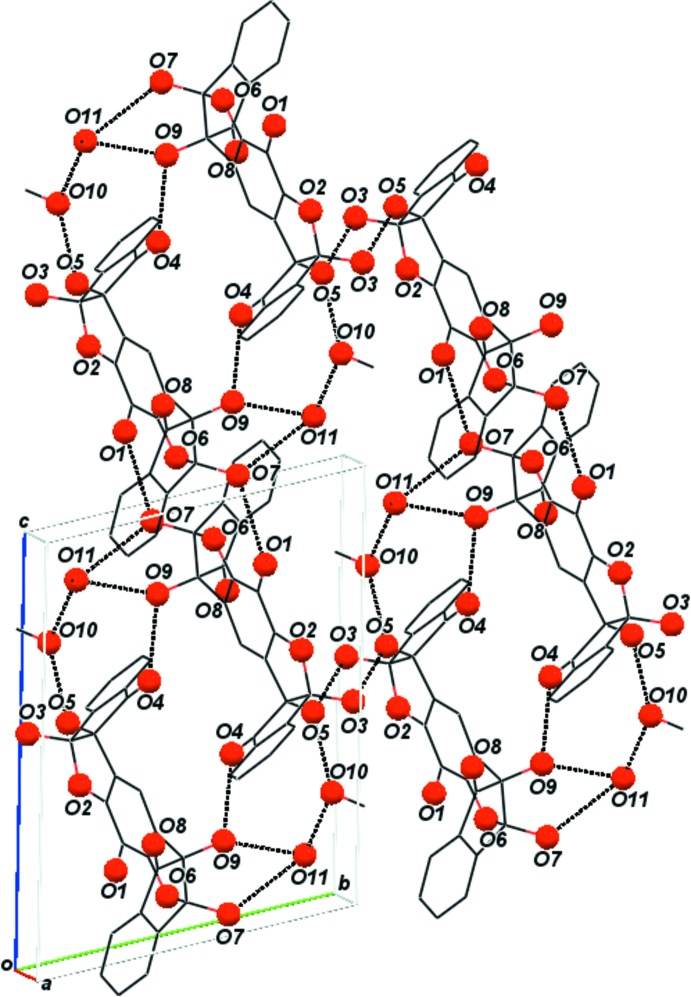
A view down nearly a axis showing the H-bonding framework of the structure.

## supplementary crystallographic information

### 1. Introduction

Kim's synthesis of a novel cavitand by a one pot reaction between phloroglucinol and ninhydrin has been extended creating a new class of bowl-shaped compounds, which we have named Vasarenes (Almog *et al.*, 2009). An isomeric form of the vasarene above, possessing a cup-shaped form has been reported in the past (Mahmood *et al.*, 2011). Under our synthetic conditions only a chair-shaped form could be obtained. Quantum chemical DFT calculations show a small energy difference (less than 0.5 kcal/mol) between these two isomers.

### 2. Results and discussion

The content of the unit cell is actually the basic feature which extended itself in 3D space. It includes the two RRRR/SSSS enanti­omers of I, and four methanol molecules. These two enanti­omers link each other by two O(9)—H···O(4) hydrogen bonds , forming a 18-member ring centered at (0.5, 0.5, 0.5). Two methanol molecules bind each other through O(10)—H···O(11) and stabilize the dimer by two extra hydrogen bonds : O(5)—H···O(10) and O(11)—H···O(9). The same applies at the opposite side of the dimer with the other two methanol molecules. A pair of O(3)—H···O(5) hydrogen bonds, centered at (0.5, 1.0, 0.5), extend the structure in the y-direction, while a pair of O(7)—H···O(1) hydrogen bonds centered at (0.5, 0.5, 1.0) extend - in the z-direction. And finally these yz layers link themselves in the x-direction by O(1)—H···O(8) hydrogen bonds, thus completing the 3D structure. Fig. 2 describes it well.

### 2.1. Synthesis and crystallization

A mixture of ninhydrin (2.00 g, 11.23 mmol) and pyrogallol (0.70 g, 5.5 mmol) in acetic acid (40 ml), was stirred at 80°C for 24h. During the reaction the mixture turned brown, and a white solid precipitated. After cooling to room temperature, the solid was filtered and washed with cold acetic acid and ether. Crystallization from methanol produced colorless crystals suitable for single crystal X-ray crystallography. Yield: 1.27g, 51%, m.p. 218°C.

### 2.2. Refinement

Hydroxyl H atoms of methanol molecules were located in a different Fourier map and refined in riding mode with distance constraint of O—H = 0.82 (1) Å, U_iso_(H) = 1.2U_eq_(O). Other H atoms were placed in calculated positions with O—H = 0.82, C—H = 0.93 (aromatic) and 0.96 Å (methyl), and refined in riding mode with U_iso_(H) = 1.2U_eq_(C) for aromatic H atoms and 1.5U_eq_(C,O) for the methyl h and other hydroxyl H atoms.

### Crystal data

**Table:**

C_24_H_14_O_9_·2CH_4_O	*Z* = 2
*M**_r_* = 510.44	*F*(000) = 532
Triclinic, *P*1	*D*_x_ = 1.421 Mg m^−^^3^
Hall symbol: -P 1	Mo *K*α radiation, λ = 0.71073 Å
*a* = 8.8243 (13) Å	Cell parameters from 3395 reflections
*b* = 10.3974 (16) Å	θ = 2.1–28.0°
*c* = 14.348 (2) Å	µ = 0.11 mm^−^^1^
α = 72.936 (3)°	*T* = 295 K
β = 74.639 (2)°	Block, colorless
γ = 75.792 (3)°	0.20 × 0.13 × 0.08 mm
*V* = 1193.3 (3) Å^3^	

### Data collection

**Table:**

Bruker SMART CCD area-detector diffractometer	3395 reflections with *I* > 2σ(*I*)
Radiation source: fine-focus sealed tube	*R*_int_ = 0.050
Graphite monochromator	θ_max_ = 28.0°, θ_min_ = 2.1°
Detector resolution: 8.36 pixels mm^-1^	*h* = −11→11
phi and ω scans	*k* = −13→13
13982 measured reflections	*l* = −18→18
5549 independent reflections	

### Refinement

**Table:**

Refinement on *F*^2^	Primary atom site location: structure-invariant direct methods
Least-squares matrix: full	Secondary atom site location: difference Fourier map
*R*[*F*^2^ > 2σ(*F*^2^)] = 0.067	Hydrogen site location: inferred from neighbouring sites
*wR*(*F*^2^) = 0.153	H atoms treated by a mixture of independent and constrained refinement
*S* = 1.01	*w* = 1/[σ^2^(*F*_o_^2^) + (0.0681*P*)^2^] where *P* = (*F*_o_^2^ + 2*F*_c_^2^)/3
5549 reflections	(Δ/σ)_max_ < 0.001
345 parameters	Δρ_max_ = 0.30 e Å^−^^3^
2 restraints	Δρ_min_ = −0.33 e Å^−^^3^

### Special details

**Table:**

Geometry. All e.s.d.'s (except the e.s.d. in the dihedral angle between two l.s. planes) are estimated using the full covariance matrix. The cell e.s.d.'s are taken into account individually in the estimation of e.s.d.'s in distances, angles and torsion angles; correlations between e.s.d.'s in cell parameters are only used when they are defined by crystal symmetry. An approximate (isotropic) treatment of cell e.s.d.'s is used for estimating e.s.d.'s involving l.s. planes.
Refinement. Refinement of *F*^2^ against ALL reflections. The weighted *R*-factor *wR* and goodness of fit *S* are based on *F*^2^, conventional *R*-factors *R* are based on *F*, with *F* set to zero for negative *F*^2^. The threshold expression of *F*^2^ > σ(*F*^2^) is used only for calculating *R*-factors(gt) *etc*. and is not relevant to the choice of reflections for refinement. *R*-factors based on *F*^2^ are statistically about twice as large as those based on *F*, and *R*- factors based on ALL data will be even larger.

### Fractional atomic coordinates and isotropic or equivalent isotropic displacement parameters (Å^2^)

**Table:**

	*x*	*y*	*z*	*U*_iso_*/*U*_eq_	
C1	0.6380 (3)	0.6944 (2)	0.75601 (17)	0.0271 (5)	
C2	0.6617 (3)	0.7483 (2)	0.65384 (17)	0.0251 (5)	
C3	0.5721 (3)	0.7275 (2)	0.59507 (16)	0.0242 (5)	
C4	0.4448 (3)	0.6583 (2)	0.63721 (16)	0.0253 (5)	
H4	0.3822	0.6465	0.5985	0.030*	
C5	0.4155 (2)	0.6076 (2)	0.74000 (16)	0.0237 (5)	
C6	0.5131 (3)	0.6235 (2)	0.79557 (16)	0.0259 (5)	
C7	0.7762 (3)	0.8563 (2)	0.49645 (17)	0.0271 (5)	
C8	0.9267 (3)	0.7759 (3)	0.44871 (18)	0.0313 (6)	
C9	1.0816 (3)	0.7894 (3)	0.4420 (2)	0.0453 (7)	
H9	1.1021	0.8543	0.4676	0.054*	
C10	1.2044 (3)	0.7035 (3)	0.3962 (2)	0.0520 (8)	
H10	1.3096	0.7102	0.3912	0.062*	
C11	1.1739 (3)	0.6066 (3)	0.3571 (2)	0.0492 (8)	
H11	1.2589	0.5511	0.3253	0.059*	
C12	1.0206 (3)	0.5921 (3)	0.3650 (2)	0.0420 (7)	
H12	1.0003	0.5264	0.3401	0.050*	
C13	0.8956 (3)	0.6787 (2)	0.41145 (18)	0.0304 (6)	
C14	0.7229 (3)	0.6854 (2)	0.42804 (16)	0.0278 (5)	
C15	0.6364 (3)	0.7924 (2)	0.48880 (16)	0.0251 (5)	
C16	0.3270 (3)	0.5121 (3)	0.91425 (17)	0.0277 (5)	
C17	0.1959 (3)	0.6094 (3)	0.96265 (17)	0.0289 (6)	
C18	0.1819 (3)	0.6424 (3)	1.05147 (19)	0.0417 (7)	
H18	0.2568	0.6003	1.0912	0.050*	
C19	0.0530 (4)	0.7400 (3)	1.0794 (2)	0.0501 (8)	
H19	0.0401	0.7623	1.1396	0.060*	
C20	−0.0564 (3)	0.8047 (3)	1.0201 (2)	0.0512 (8)	
H32	−0.1408	0.8712	1.0402	0.061*	
C21	−0.0430 (3)	0.7726 (3)	0.9319 (2)	0.0425 (7)	
H21	−0.1171	0.8163	0.8918	0.051*	
C22	0.0840 (3)	0.6733 (3)	0.90383 (18)	0.0315 (6)	
C23	0.1240 (3)	0.6198 (3)	0.81479 (17)	0.0287 (6)	
C24	0.2903 (3)	0.5289 (2)	0.80993 (16)	0.0258 (5)	
C25	0.7395 (5)	1.0380 (4)	0.2088 (3)	0.0889 (13)	
H25A	0.7409	1.0880	0.2551	0.133*	
H25B	0.6977	1.0999	0.1534	0.133*	
H25C	0.8462	0.9938	0.1854	0.133*	
C26	0.6111 (6)	0.1071 (5)	0.8833 (4)	0.1277 (19)	
H26A	0.6181	0.1204	0.9453	0.192*	
H26B	0.6683	0.1680	0.8290	0.192*	
H26C	0.6568	0.0142	0.8803	0.192*	
O1	0.72441 (18)	0.7100 (2)	0.81690 (12)	0.0381 (5)	
H1O	0.8184	0.7067	0.7882	0.057*	
O2	0.77457 (18)	0.82605 (17)	0.60350 (12)	0.0322 (4)	
O3	0.76792 (19)	0.99359 (17)	0.45672 (13)	0.0354 (4)	
H3O	0.6807	1.0345	0.4802	0.053*	
O4	0.6535 (2)	0.62199 (19)	0.39918 (13)	0.0409 (5)	
O5	0.51468 (19)	0.88543 (17)	0.44504 (12)	0.0332 (4)	
H5O	0.5481	0.9108	0.3848	0.050*	
O6	0.47605 (18)	0.56300 (18)	0.89531 (11)	0.0317 (4)	
O7	0.3494 (2)	0.37741 (17)	0.96603 (12)	0.0387 (5)	
H7O	0.3369	0.3731	1.0255	0.058*	
O8	0.0433 (2)	0.6403 (2)	0.75343 (13)	0.0455 (5)	
O9	0.2920 (2)	0.40219 (17)	0.79333 (12)	0.0381 (5)	
H9O	0.3109	0.4088	0.7332	0.057*	
O10	0.6448 (4)	0.9415 (3)	0.25526 (18)	0.1024 (11)	
H10O	0.604 (5)	0.909 (4)	0.224 (3)	0.123*	
O11	0.4543 (4)	0.1337 (3)	0.8764 (2)	0.0857 (8)	
H11O	0.420 (5)	0.2163 (14)	0.873 (3)	0.103*	

### Atomic displacement parameters (Å^2^)

**Table:**

	*U*^11^	*U*^22^	*U*^33^	*U*^12^	*U*^13^	*U*^23^
C1	0.0177 (12)	0.0415 (15)	0.0265 (13)	−0.0033 (10)	−0.0055 (10)	−0.0161 (11)
C2	0.0164 (11)	0.0343 (14)	0.0257 (13)	−0.0045 (10)	−0.0013 (9)	−0.0121 (11)
C3	0.0193 (11)	0.0301 (13)	0.0236 (12)	−0.0020 (10)	−0.0040 (9)	−0.0094 (10)
C4	0.0207 (12)	0.0355 (14)	0.0236 (12)	−0.0055 (10)	−0.0068 (10)	−0.0109 (10)
C5	0.0160 (11)	0.0313 (13)	0.0249 (12)	−0.0036 (10)	−0.0035 (9)	−0.0098 (10)
C6	0.0189 (12)	0.0376 (14)	0.0189 (12)	−0.0010 (10)	−0.0012 (9)	−0.0096 (10)
C7	0.0257 (13)	0.0330 (14)	0.0245 (13)	−0.0107 (10)	−0.0005 (10)	−0.0097 (11)
C8	0.0279 (13)	0.0345 (14)	0.0280 (13)	−0.0067 (11)	0.0000 (10)	−0.0065 (11)
C9	0.0314 (15)	0.0555 (19)	0.0541 (18)	−0.0093 (13)	−0.0039 (13)	−0.0244 (15)
C10	0.0236 (14)	0.067 (2)	0.063 (2)	−0.0049 (14)	0.0007 (14)	−0.0242 (17)
C11	0.0341 (16)	0.0509 (19)	0.0534 (19)	0.0045 (14)	0.0028 (14)	−0.0195 (15)
C12	0.0406 (17)	0.0414 (16)	0.0436 (16)	−0.0045 (13)	−0.0008 (13)	−0.0193 (13)
C13	0.0289 (14)	0.0326 (14)	0.0276 (13)	−0.0054 (11)	−0.0021 (10)	−0.0080 (11)
C14	0.0338 (14)	0.0323 (14)	0.0171 (12)	−0.0117 (11)	0.0002 (10)	−0.0058 (10)
C15	0.0227 (12)	0.0317 (13)	0.0222 (12)	−0.0052 (10)	−0.0047 (10)	−0.0085 (10)
C16	0.0219 (12)	0.0394 (15)	0.0239 (12)	−0.0086 (10)	−0.0047 (10)	−0.0083 (11)
C17	0.0240 (13)	0.0385 (14)	0.0253 (13)	−0.0118 (11)	0.0016 (10)	−0.0106 (11)
C18	0.0420 (16)	0.0548 (18)	0.0312 (15)	−0.0054 (14)	−0.0071 (12)	−0.0182 (13)
C19	0.0463 (18)	0.071 (2)	0.0414 (17)	−0.0076 (16)	−0.0050 (14)	−0.0331 (16)
C20	0.0318 (16)	0.066 (2)	0.061 (2)	0.0022 (14)	−0.0057 (14)	−0.0372 (17)
C21	0.0236 (14)	0.0600 (19)	0.0489 (17)	−0.0031 (13)	−0.0084 (12)	−0.0240 (15)
C22	0.0191 (12)	0.0465 (16)	0.0329 (14)	−0.0104 (11)	−0.0008 (10)	−0.0160 (12)
C23	0.0168 (12)	0.0465 (16)	0.0245 (13)	−0.0134 (11)	−0.0022 (10)	−0.0069 (11)
C24	0.0243 (12)	0.0344 (14)	0.0209 (12)	−0.0078 (10)	−0.0024 (10)	−0.0100 (10)
C25	0.134 (4)	0.069 (3)	0.058 (2)	−0.043 (3)	0.014 (2)	−0.017 (2)
C26	0.081 (4)	0.129 (4)	0.168 (5)	−0.004 (3)	−0.036 (4)	−0.031 (4)
O1	0.0209 (9)	0.0731 (13)	0.0273 (9)	−0.0157 (9)	−0.0045 (7)	−0.0174 (9)
O2	0.0262 (9)	0.0483 (11)	0.0274 (9)	−0.0165 (8)	−0.0019 (7)	−0.0124 (8)
O3	0.0291 (10)	0.0322 (10)	0.0437 (11)	−0.0098 (8)	−0.0002 (8)	−0.0107 (8)
O4	0.0412 (11)	0.0536 (12)	0.0368 (10)	−0.0186 (9)	0.0001 (8)	−0.0237 (9)
O5	0.0283 (9)	0.0418 (10)	0.0269 (9)	−0.0046 (8)	−0.0067 (7)	−0.0053 (8)
O6	0.0216 (9)	0.0537 (11)	0.0208 (9)	−0.0113 (8)	−0.0050 (7)	−0.0064 (8)
O7	0.0511 (12)	0.0408 (11)	0.0235 (9)	−0.0113 (9)	−0.0083 (8)	−0.0041 (8)
O8	0.0255 (10)	0.0782 (15)	0.0389 (11)	−0.0064 (9)	−0.0104 (8)	−0.0225 (10)
O9	0.0518 (12)	0.0409 (11)	0.0273 (9)	−0.0193 (9)	−0.0059 (8)	−0.0099 (8)
O10	0.159 (3)	0.127 (2)	0.0380 (14)	−0.096 (2)	−0.0015 (16)	−0.0037 (15)
O11	0.083 (2)	0.0627 (17)	0.119 (2)	0.0077 (15)	−0.0398 (17)	−0.0354 (17)

### Geometric parameters (Å, º)

**Table:**

C1—O1	1.367 (3)	C16—C17	1.502 (3)
C1—C6	1.379 (3)	C16—C24	1.566 (3)
C1—C2	1.386 (3)	C17—C18	1.382 (3)
C2—O2	1.356 (3)	C17—C22	1.386 (3)
C2—C3	1.388 (3)	C18—C19	1.384 (4)
C3—C4	1.388 (3)	C18—H18	0.9300
C3—C15	1.492 (3)	C19—C20	1.375 (4)
C4—C5	1.389 (3)	C19—H19	0.9300
C4—H4	0.9300	C20—C21	1.371 (4)
C5—C6	1.384 (3)	C20—H32	0.9300
C5—C24	1.507 (3)	C21—C22	1.385 (3)
C6—O6	1.369 (3)	C21—H21	0.9300
C7—O3	1.364 (3)	C22—C23	1.466 (3)
C7—O2	1.472 (3)	C23—O8	1.215 (3)
C7—C8	1.497 (3)	C23—C24	1.534 (3)
C7—C15	1.579 (3)	C24—O9	1.402 (3)
C8—C13	1.383 (3)	C25—O10	1.369 (4)
C8—C9	1.384 (3)	C25—H25A	0.9600
C9—C10	1.377 (4)	C25—H25B	0.9600
C9—H9	0.9300	C25—H25C	0.9600
C10—C11	1.395 (4)	C26—O11	1.367 (5)
C10—H10	0.9300	C26—H26A	0.9600
C11—C12	1.369 (4)	C26—H26B	0.9600
C11—H11	0.9300	C26—H26C	0.9600
C12—C13	1.395 (3)	O1—H1O	0.8200
C12—H12	0.9300	O3—H3O	0.8200
C13—C14	1.467 (3)	O5—H5O	0.8200
C14—O4	1.214 (3)	O7—H7O	0.8194
C14—C15	1.543 (3)	O9—H9O	0.8194
C15—O5	1.405 (3)	O10—H10O	0.820 (10)
C16—O7	1.373 (3)	O11—H11O	0.828 (10)
C16—O6	1.467 (3)		
			
O1—C1—C6	120.2 (2)	C17—C16—C24	105.47 (18)
O1—C1—C2	125.2 (2)	C18—C17—C22	120.5 (2)
C6—C1—C2	114.6 (2)	C18—C17—C16	128.1 (2)
O2—C2—C1	122.4 (2)	C22—C17—C16	111.3 (2)
O2—C2—C3	114.8 (2)	C17—C18—C19	118.0 (3)
C1—C2—C3	122.9 (2)	C17—C18—H18	121.0
C4—C3—C2	121.2 (2)	C19—C18—H18	121.0
C4—C3—C15	130.4 (2)	C20—C19—C18	121.4 (3)
C2—C3—C15	108.38 (19)	C20—C19—H19	119.3
C3—C4—C5	116.7 (2)	C18—C19—H19	119.3
C3—C4—H4	121.6	C21—C20—C19	120.9 (3)
C5—C4—H4	121.6	C21—C20—H32	119.5
C6—C5—C4	120.4 (2)	C19—C20—H32	119.5
C6—C5—C24	108.21 (19)	C20—C21—C22	118.3 (3)
C4—C5—C24	131.3 (2)	C20—C21—H21	120.9
O6—C6—C1	121.4 (2)	C22—C21—H21	120.9
O6—C6—C5	114.6 (2)	C21—C22—C17	121.0 (2)
C1—C6—C5	124.0 (2)	C21—C22—C23	129.0 (2)
O3—C7—O2	109.01 (18)	C17—C22—C23	110.0 (2)
O3—C7—C8	111.65 (19)	O8—C23—C22	127.9 (2)
O2—C7—C8	107.93 (19)	O8—C23—C24	123.6 (2)
O3—C7—C15	116.6 (2)	C22—C23—C24	108.51 (19)
O2—C7—C15	105.89 (17)	O9—C24—C5	115.82 (18)
C8—C7—C15	105.35 (19)	O9—C24—C23	112.00 (18)
C13—C8—C9	121.3 (2)	C5—C24—C23	110.43 (19)
C13—C8—C7	111.8 (2)	O9—C24—C16	111.61 (19)
C9—C8—C7	126.9 (2)	C5—C24—C16	102.36 (17)
C10—C9—C8	117.9 (3)	C23—C24—C16	103.53 (17)
C10—C9—H9	121.1	O10—C25—H25A	109.5
C8—C9—H9	121.1	O10—C25—H25B	109.5
C9—C10—C11	121.2 (3)	H25A—C25—H25B	109.5
C9—C10—H10	119.4	O10—C25—H25C	109.5
C11—C10—H10	119.4	H25A—C25—H25C	109.5
C12—C11—C10	120.9 (3)	H25B—C25—H25C	109.5
C12—C11—H11	119.6	O11—C26—H26A	109.5
C10—C11—H11	119.6	O11—C26—H26B	109.5
C11—C12—C13	118.2 (3)	H26A—C26—H26B	109.5
C11—C12—H12	120.9	O11—C26—H26C	109.5
C13—C12—H12	120.9	H26A—C26—H26C	109.5
C8—C13—C12	120.6 (2)	H26B—C26—H26C	109.5
C8—C13—C14	110.5 (2)	C1—O1—H1O	109.5
C12—C13—C14	128.9 (2)	C2—O2—C7	108.05 (16)
O4—C14—C13	128.1 (2)	C7—O3—H3O	109.5
O4—C14—C15	123.4 (2)	C15—O5—H5O	109.5
C13—C14—C15	108.41 (19)	C6—O6—C16	107.45 (16)
O5—C15—C3	110.68 (19)	C16—O7—H11O	116.6 (9)
O5—C15—C14	111.54 (18)	C16—O7—H7O	109.4
C3—C15—C14	112.18 (19)	H11O—O7—H7O	134.0
O5—C15—C7	115.65 (19)	C24—O9—H11O	120.8 (10)
C3—C15—C7	102.74 (18)	C24—O9—H9O	109.2
C14—C15—C7	103.64 (18)	H11O—O9—H9O	112.8
O7—C16—O6	108.50 (18)	C25—O10—H5O	124.0
O7—C16—C17	117.07 (19)	C25—O10—H10O	123 (3)
O6—C16—C17	107.07 (19)	H5O—O10—H10O	111.0
O7—C16—C24	111.54 (19)	C26—O11—H11O	108 (3)
O6—C16—C24	106.64 (17)		

### Hydrogen-bond geometry (Å, º)

Cg is the centroid of the C1–C6 benzene ring.

**Table:**

*D*—H···*A*	*D*—H	H···*A*	*D*···*A*	*D*—H···*A*
O1—H1*O*···O8^i^	0.82	1.92	2.702 (2)	160
O3—H3*O*···O5^ii^	0.82	1.91	2.725 (2)	171
O5—H5*O*···O10	0.82	1.80	2.610 (3)	171
O7—H7*O*···O1^iii^	0.82	2.14	2.916 (2)	158
O9—H9*O*···O4^iv^	0.82	1.95	2.751 (2)	166
O10—H10*O*···O11^iv^	0.82 (1)	1.85 (2)	2.650 (4)	163 (5)
O11—H11*O*···O7	0.83 (1)	2.31 (3)	3.010 (3)	142 (4)
O11—H11*O*···O9	0.83 (1)	2.15 (3)	2.873 (3)	145 (4)
C18—H18···O6^iii^	0.93	2.56	3.370 (3)	146
C21—H21···O1^v^	0.93	2.57	3.231 (3)	129
C11—H11···*Cg*^vi^	0.93	2.63	3.501 (3)	156

Symmetry codes: (i) *x*+1, *y*, *z*; (ii) −*x*+1, −*y*+2, −*z*+1; (iii) −*x*+1, −*y*+1, −*z*+2; (iv) −*x*+1, −*y*+1, −*z*+1; (v) *x*−1, *y*, *z*; (vi) −*x*+2, −*y*+1, −*z*+1.
